# Celebrating the legacy of Prof. Ovidiu Alexandru Bajenaru

**DOI:** 10.25122/jml-2022-1002

**Published:** 2022-02

**Authors:** Dafin Muresanu, Cristina Tiu, Bogdan Popescu

**Affiliations:** 1. Department of Neuroscience, Iuliu Hatieganu University of Medicine and Pharmacy, Cluj-Napoca, Romania; 2. RoNeuro Institute for Neurological Research and Diagnostic, Cluj-Napoca, Romania; 3. Department of Clinical Neurosciences, Carol Davila University of Medicine and Pharmacy, Bucharest, Romania; 4. Stroke Unit, Department of Neurology, University Emergency Hospital, Bucharest, Romania; 5. Department of Neurology, Colentina Clinical Hospital, Bucharest, Romania

We find ourselves almost one and a half year after the timely passing of our friend and mentor, professor **Dr. Ovidiu Alexandru Băjenaru**. A brilliant neurologist, researcher, and teacher, he leaves behind a profound legacy of perhaps the most influential contemporary figure in Romanian neurology.

We remember and cherish some of his most significant achievements over his long and fruitful career. Professor Băjenaru is the founding president of the Romanian Society of Neurology (RSN), an organization he formally led for 12 years. In this position, he contributed decisively to advancing science and education in our field. He spearheaded the development of a vision for clinical neurology in our country, based on the conceptual changes determined by colossal advances in neuroscience and modern medical technologies, and integrated using traditional clinical training.

Professor Ovidiu Băjenaru represented Romania at top-notch medical forums. He was Romania's official representative at the World Federation of Neurology (since 2001), the European Stroke Organization, and the European Union of Medical Specialists (Board of Neurology permanent representative of Romania since 2008). Dr. Băjenaru was also a corresponding member of the Romanian Academy (since 2016), as well as a member of the Romanian Group of Brain Research (since 2016), member of the Bureau of Medical Services (since 2018), member of the Academy of Medical Sciences (since 2013).

His primary areas of interest were neurodegenerative diseases (particularly Parkinson's disease and Alzheimer's disease), muscular dystonia and other motor behavior abnormalities, cerebrovascular diseases, multiple sclerosis, and sleep pathology in neurological disorders and medical conditions – all fields in which he became a leading authority. His scientific activity includes over 500 presentations at national and international events, as well as over 50 international multicentric clinical trials as principal investigator.

Professor Băjenaru coordinated the interdisciplinary medical group for the treatment by deep brain stimulation for Parkinson's disease (neurology, neurosurgery, medical imaging) from the Bucharest Emergency University Hospital (since 2006), the first Romanian medical group for the interventional treatment of epilepsy (since 2009), and the national health programs for neurological disorders of the National Health Insurance House and the Romanian Ministry of Health (since 2014). Between 2008–2014, he was the President of the Neurology and Pediatric Neurology Commission at the Romanian Ministry of Health.

Among his original scientific contributions, as part of his doctoral thesis defended in 1993, he introduced for the first time in Romania the concept of incipient cognitive impairment, referring to patients with cerebrovascular and coronary diseases who develop neurocognitive disorders, as a condition preceding the onset of dementia. He also promoted the implementation of systematic neurocognitive evaluation for patients with neurological and vascular diseases, as well as their treatment, in the current pursuit/activity of the neurologists in Romania. Dr. Băjenaru conducted the first brain connectomics study in the context of a neurological condition (Parkinson's Disease) in Romania.

Under the coordination of professor Băjenaru, a modern school of clinicians and researchers in neurosciences was born, forming several professors and heads of clinics and departments in Bucharest. His efforts were instrumental in the training and education of future generations of doctors. In this regard, he consistently supported and encouraged access to an impressive number of research grants and training courses in major European centers in close collaboration with various European companies.

Thus, in the spirit of this great personality, FSNR announced at the beginning of 2021 the opening of the application procedure for the “Professor Ovidiu Alexandru Băjenaru” educational scholarship for resident doctors and neurologists. The scholarship applies for the next academic year (the scholarship is usually awarded in July).

The main objective of the scholarship is to support resident doctors and neurologists in their professional and/or didactic development by carrying out internships in various neurology clinics abroad.

He was an influencing neurologist, a fantastic colleague, and a dear friend. It is a great loss for all of us that he has passed away at such a young age. Although he is no longer with us, his powerful impact on our field will last for a very long time. We, the medical specialists from the Romanian scientific and academic environment, will continue and try to further develop the work professor Bajenaru has left behind.

